# Biliary network linkage using a side-hole-modified metal stent for concurrent endoscopic ultrasound-guided hepaticoduodenostomy

**DOI:** 10.1055/a-2789-2231

**Published:** 2026-02-27

**Authors:** Hiroki Koda, Kazuo Hara, Tomoki Ogata, Shin Haba, Takamichi Kuwahara, Nozomi Okuno, Shimpei Matsumoto

**Affiliations:** 1 538357 Department of Gastroenterology, Aichi Cancer Center, Nagoya, Japan

Endoscopic ultrasound (EUS)-guided stent bridging and related techniques enable the traversal of separated intrahepatic ducts in complex malignant hilar obstruction 1 2 3 . We refer to the intentional creation of a functional communication between anatomically separated ducts as EUS-guided biliary network linkage (EUS-BNL), a conceptual framework emphasizing the reconstruction of an internal drainage pathway. Side holes manually created on a fully covered self-expandable metal stent (FCSEMS) may help prevent occlusion of intrahepatic side branches during drainage 4 . In this case, such a modified FCSEMS enabled both BNL and EUS-guided hepaticoduodenostomy (EUS-HDS) to be achieved with the same single removable stent, facilitating reintervention ( Video 1 ).

An EUS-guided approach using a side-hole-modified fully covered metal stent to achieve EUS-guided biliary network linkage (EUS-BNL) and hepaticoduodenostomy with the same single stent, enabling multisegmental drainage in complex hilar obstruction. EUS, endoscopic ultrasound.Video 1

**Fig. 1:**
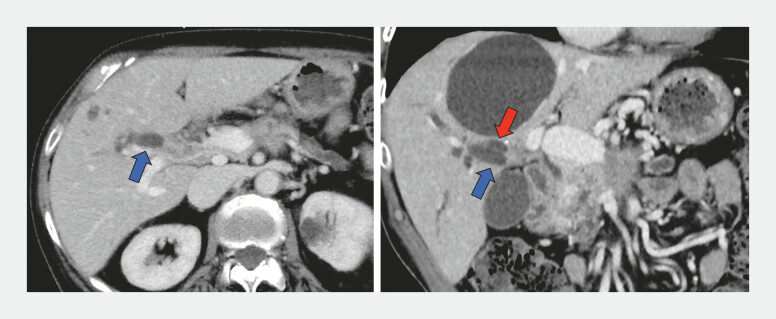
Computed tomography demonstrates the complete separation of the right anterior (blue arrow) and posterior (red arrow) segmental bile ducts caused by peritoneal dissemination from pancreatic cancer infiltrating the hepatic hilum.

**Fig. 2:**
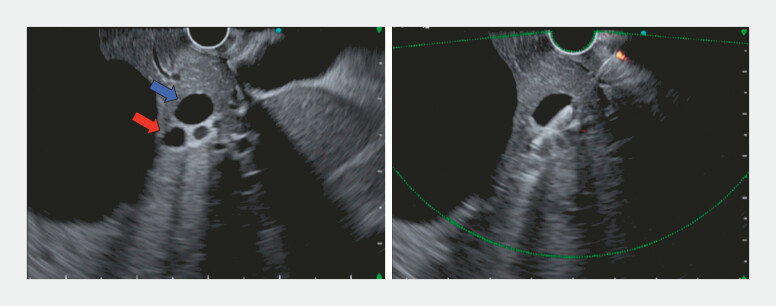
The anterior (blue arrow) and posterior (red arrow) segmental bile ducts were skewered under EUS guidance to obtain sequential access for endoscopic ultrasound-guided biliary network linkage (EUS-BNL).

A 76-year-old woman presented with malignant hilar obstruction caused by peritoneal dissemination of pancreatic cancer, resulting in the complete separation of the right anterior and posterior ducts ( Fig. 1 ). Because further anatomic complexity was expected, EUS-HDS was selected as the primary drainage route. From the duodenal bulb, convex EUS (EG740UT; FUJIFILM) demonstrated both ducts aligned along the intended access path, enabling sequential traversal. A 19-gauge needle (EZ Shot 3 Plus; Olympus) was advanced through hepatic parenchyma to skewer both ducts ( Fig. 2 ). After cholangiography confirmed positioning, a guidewire was inserted into B7, and the tract was dilated using a drill dilator.

**Fig. 3:**
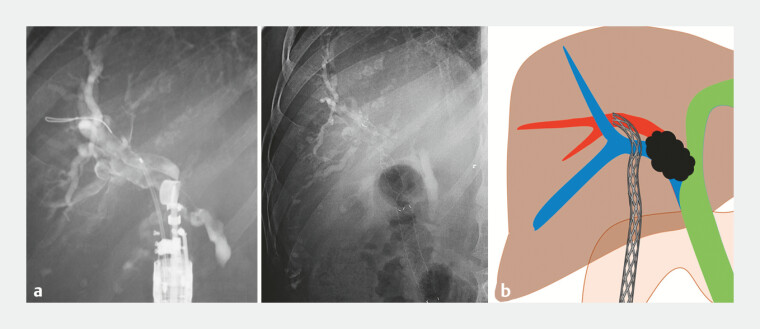
A single metal stent placed via biliary skewering establishes interductal linkage consistent with the EUS-BNL concept and simultaneously serves as the hepaticoduodenostomy stent. **a** A fluoroscopic image. **b** Schematic illustration. EUS-BNL, endoscopic ultrasound-guided biliary network linkage.

**Fig. 4:**
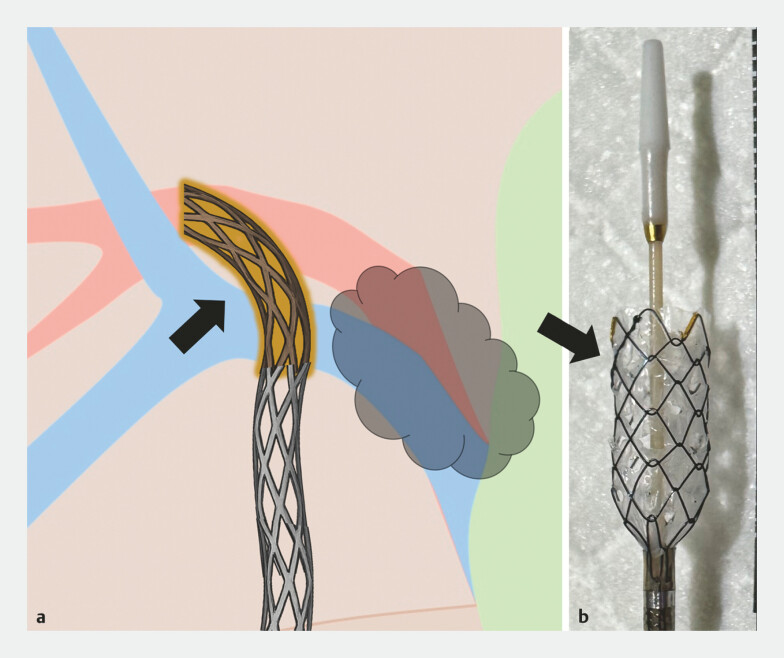
The blurred segment (arrow) represents manually created side holes in the stent mesh, enabling the effective drainage of the anterior duct while using the same stent for concurrent EUS-HDS. **a** Schematic illustration. **b** An image showing the portion of the stent where multiple side holes were created. EUS-HDS, endoscopic ultrasound-guided hepaticoduodenostomy.

A single FCSEMS (HANAROSTENT Biliary Benefit, 8 mm × 12 cm) was deployed from the posterior duct into the duodenum, establishing interductal linkage consistent with the EUS-BNL concept ( Fig. 3 ). The side-hole-modified distal segment was positioned to overlap the anterior duct, providing drainage while the same stent concurrently served as the HDS stent ( Fig. 4 ). Because the stent is fully covered and removable, reintervention can be performed as needed.

This case demonstrates that EUS-BNL offers an effective strategy for multisegmental drainage and that side-hole modification enables concurrent EUS-HDS with the same single stent in complex hilar obstruction.

Endoscopy_UCTN_Code_TTT_1AR_2AI
